# Indexation of extravascular lung water in unselected adult patients with and without mechanical ventilation: a prospective study in 50 patients with 843 transpulmonary thermodilutions

**DOI:** 10.1186/cc10852

**Published:** 2012-03-20

**Authors:** W Huber, B Saugel, D Paradellis, J Hoellthaler, V Phillip, C Schultheiss, P Thies, U Mayr, A Herrmann, RM Schmid

**Affiliations:** 1 Klinikum Rechts der Isar, Technischen Universität München, Munich, Germany

## Introduction

Extravascular lung water (EVLW) has been indexed to actual BW (BW-act), termed the EVLW index (ELWI). Since in obese patients indexation to BW-act might inappropriately diminish the indexed ELWI-act, ELWI indexed to predicted BW (ELWI-pred) has been introduced. Indexation of EVLW to height might be superior to ELWI-pred/-act. Recent data in a selected collective of ARDS patients suggest that indexation to height might improve the predictive capabilities of ELWI regarding pO_2_/FiO_2_. We aimed to investigate which indexation of EVLW provides the best association of ELWI and pO_2_/FiO_2 _in patients without pulmonary impairment or without ventilation.

## Methods

In 50 consecutive ICU patients with PiCCO monitoring, 843 triplicate measurements of EVLW and simultaneous blood gas analysis were performed. The endpoint was prediction of pO_2_/FiO_2 _<200 mmHg and other critical thresholds provided by unindexed EVLW as well as ELWI indexed to ideal BW, adjusted BW, BMI, body surface area, height and total lung capacity.

## Results

Measurements in patients without pulmonary impairment 463/843 (54.9%); acute 188/843 (22.3%), chronic 106/843 (12.6%), and both acute and chronic pulmonary disease 86/843 (10.2%). Mechanical ventilation in 458/843 (54.3%) measurements. The largest ROC AUCs regarding pO_2_/FiO_2 _<200 mmHg were found for ELWI-height (AUC 0.658; 95% CI 0.554 to 0.735) and EVLW (0.655; 95% CI 0.544 to 0.732), the lowest AUC for ELWI-act (0.629; 95% CI 0.514 to 0.742). Similarly ELWI-height and unindexed EVLW provided the largest ROC AUCs regarding pO_2_/FiO_2 _>300 mmHg (0.659 and 0.657), normal pO_2_/FiO_2 _(>381 mmHg; 0.665 and 0.657) and acute and/or chronic pulmonary impairment (0.622 and 0.625). All these associations were significant with *P *< 0.001. Among patients with pulmonary impairment, first values of ELWI-height and EVLW provided the largest ROC AUCs regarding mortality (0.815 and 0.815; *P *= 0.016) compared to ELWI-act (0.694; *P *= 0.136) and APACHE II score (0.792; *P *= 0.025).

## Conclusion

Indexation to BW-act results in reduced predictive capabilities compared to no indexation at all. ELWI-pred performs slightly better than ELWI-act, but our data do not support that ELWI-pred is superior to no indexation at all in adult ICU patients. In this unselected and prospectively evaluated collective, the highest predictive capabilities regarding several predefined thresholds were found for ELWI-height.

